# Phage-mediated Dispersal of Biofilm and Distribution of Bacterial Virulence Genes Is Induced by Quorum Sensing

**DOI:** 10.1371/journal.ppat.1004653

**Published:** 2015-02-23

**Authors:** Friederike S. Rossmann, Tomas Racek, Dominique Wobser, Jacek Puchalka, Elaine M. Rabener, Matthias Reiger, Antoni P. A. Hendrickx, Ann-Kristin Diederich, Kirsten Jung, Christoph Klein, Johannes Huebner

**Affiliations:** 1 Division of Infectious Diseases, Department of Medicine, University Hospital, Freiburg, Germany; 2 Faculty of Biology, Albert-Ludwigs-University, Freiburg, Germany; 3 Department of Pediatrics, Dr. von Hauner Children´s Hospital, Ludwig-Maximilians University of Munich, Munich, Germany; 4 German Center for Infection Research (DZIF), Partnersite Munich, Munich, Germany; 5 Department of Biology I, Microbiology, Munich Center for Integrated Protein Science, Ludwig-Maximilians University of Munich, Munich, Germany; 6 Department of Medical Microbiology, University Medical Center, Utrecht, The Netherlands; The University of Texas Health Science Center at San Antonio, UNITED STATES

## Abstract

The microbiome and the phage meta-genome within the human gut are influenced by antibiotic treatments. Identifying a novel mechanism, here we demonstrate that bacteria use the universal communication molecule AI-2 to induce virulence genes and transfer them via phage release. High concentrations (i.e. 100 μM) of AI-2 promote dispersal of bacteria from already established biofilms, and is associated with release of phages capable of infecting other bacteria. *Enterococcus faecalis* V583ΔABC harbours 7 prophages in its genome, and a mutant deficient in one of these prophages (i.e. prophage 5) showed a greatly reduced dispersal of biofilm. Infection of a probiotic *E. faecalis* strain without lytic prophages with prophage 5 resulted in increased biofilm formation and also in biofilm dispersal upon induction with AI-2. Infection of the probiotic *E. faecalis* strain with phage-containing supernatants released through AI-2 from *E. faecalis* V583ΔABC resulted in a strong increase in pathogenicity of this strain. The polylysogenic probiotic strain was also more virulent in a mouse sepsis model and a rat endocarditis model. Both AI-2 and ciprofloxacin lead to phage release, indicating that conditions in the gastrointestinal tract of hospitalized patients treated with antibiotics might lead to distribution of virulence genes to apathogenic enterococci and possibly also to other commensals or even to beneficial probiotic strains.

## Author Summary

All higher organisms live in intimate contact with bacteria and viruses in their direct environment. Some of these bacteria in our gut can switch between being harmless commensals and causing severe and sometimes lethal infections. This involves a tight regulation of the mechanisms needed to initially colonize and later to harm the host. Here we describe a novel mechanism by which phages (i.e. viruses that infect bacteria) contribute to virulence in commensal gut bacteria. Our results show that bacteria "sense" the number of bacteria present at any given moment through a process called quorum sensing and this provides them with the information needed to assess the specific step during the infectious process. At late stages of infection bacteria are usually present in high numbers, and at this point release viruses that can infect nearby bacteria and transfer genes that are needed to cause infection, thereby enabling previously harmless bacteria to become dangerous pathogens.

## Introduction

Phages profoundly influence ecological networks in bacterial communities by both serving as reservoirs of genetic diversity and by acting as predators of susceptible bacterial strains [1]. Although it has been known for some time that phages are important vectors for transmission of virulence and antibiotic resistance genes [1–4], the extent of their involvement in the colonization of hospitalized patients has only recently been studied [2,5]. It has been suggested that some clinical isolates may lack a functional CRISPR-CAS system leading to more adaptable strains that are able to acquire new virulence factors [6]. However, the precise mechanisms and triggers of phage release and the dynamics of infection of gut commensals is still poorly understood [1,2,7,8].

In the process of quorum sensing, bacteria detect secreted signaling molecules from the same or even different species, and respond by up- or down-regulation of genes to adapt to their environment. Autoinducer-2 (AI-2), a universal bacterial communication molecule, has been identified and studied extensively [9]. This molecule (4,5-dihydroxy-2,3-pentanedione) is produced by many bacterial species, including enterococci, and has previously been shown to be involved in the regulation of biofilm formation [10]. Generally, the biofilm mode of growth is used by bacteria to establish an infection, while later stages of biofilm maturation involve the detachment (i.e. dispersal) of a sub-fraction of bacteria from the biofilm, resulting in the dissemination of infection [11]. Enterococci are gut commensals and some *Enterococcus faecalis s*trains are strong biofilm producers, leading to gastrointestinal colonization [12] and causing severe biofilm-related infections (i.e., endocarditis, central-venous-catheter infections, or implant infections) [13–16]. Patients undergoing antineoplastic chemotherapy and receiving multiple courses of broad-spectrum antibiotics are highly susceptible to enterococcal bloodstream infections originating from colonizing bacteria in the colon [17].

Here we investigate the regulation of enterococcal pathogenicity by AI-2 and transfer of virulence genes to a probiotic *E. faecalis* strain by phage release.

## Results

**Fig 1:**
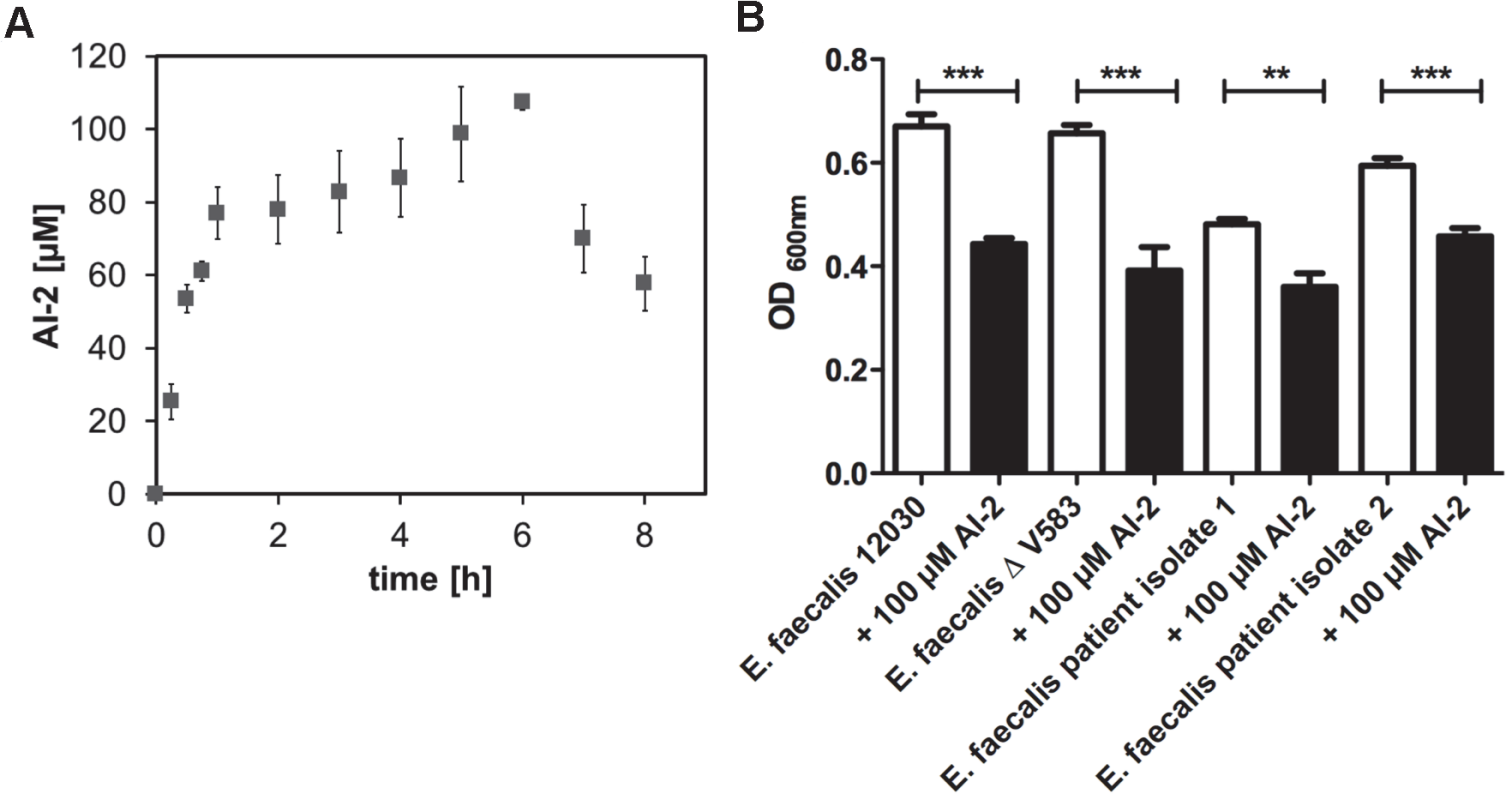
Time course of AI-2 production during growth of *E. faecalis* V583ΔABC (A) and biofilm formation of *E. faecalis* 12030, V583 ΔABC and two clinical isolates of *E. faecalis* (B). (**A)** AI-2 concentration in the supernatants was determined by a FRET-based assay for the growing culture (grey rectangle), values are shown on the left Y axis. The X-axis shows the time in hours. **(B)** A microtiter plate biofilm assay was done with staining of the attached bacteria by crystal violet. Biofilm formation was measured in absence or presence of 100 μM AI-2. Absorbance was measured at 600 nm. Statistical analysis was done by ANOVA (p<0.001) with Dunnett post test. * indicates p<0.05, ** p<0.01. Error bars represent standard error of the mean.

Since bacteria use AI-2 for quorum sensing as lingua franca, we first wanted to know whether *E. faecalis* produces AI-2. Therefore we analyzed the time course and the maximum amount of secreted AI-2. The concentration was measured in supernatants of a growing culture of *E. faecalis* V583ΔABC [18] over 8 hours (Fig. 1A). At 5 to 6 hours the AI-2 concentration peaked at a maximum of 108 μM (+/- 2 μM) and then slowly decreased. Increased production of AI-2 correlates with the exponential growth of bacteria and increase in cell density, both characteristics of quorum sensing. No significantly different AI-2 production was observed in the *pp5-* mutant (S1 Fig.). When exogenous AI-2 at a concentration of 100 μM was added to a growing culture, initial values reached 100 μM and increased up to 130 μM within the first hour. Afterwards the AI-2 concentration drops over time, indicating that bacteria sense the AI-2 in the culture environment and stop endogenous production accordingly. When no exogenous AI-2 is added, the amount produced in the culture reaches about 100 μM after 6 hours (S2 Fig.).

We next assessed whether AI-2 has an effect on biofilm production. As 100 μM AI-2 was the maximum amount produced by *E. faecalis*, we supplemented *E. faecalis* cultures with 100 μM AI-2 and observed a decrease in biofilm formation for all tested strains (Fig. 1B). The decrease in biofilm formation for *E. faecalis* V583ΔABC was corroborated by scanning electron microscopy showing less biofilm when 100 μM AI-2 was added in comparison to the cultures without supplemental AI-2 (S3 Fig.). The addition of AI-2 had no influence on the overall growth of *E. faecalis* (S4 Fig.).

**Fig 2:**
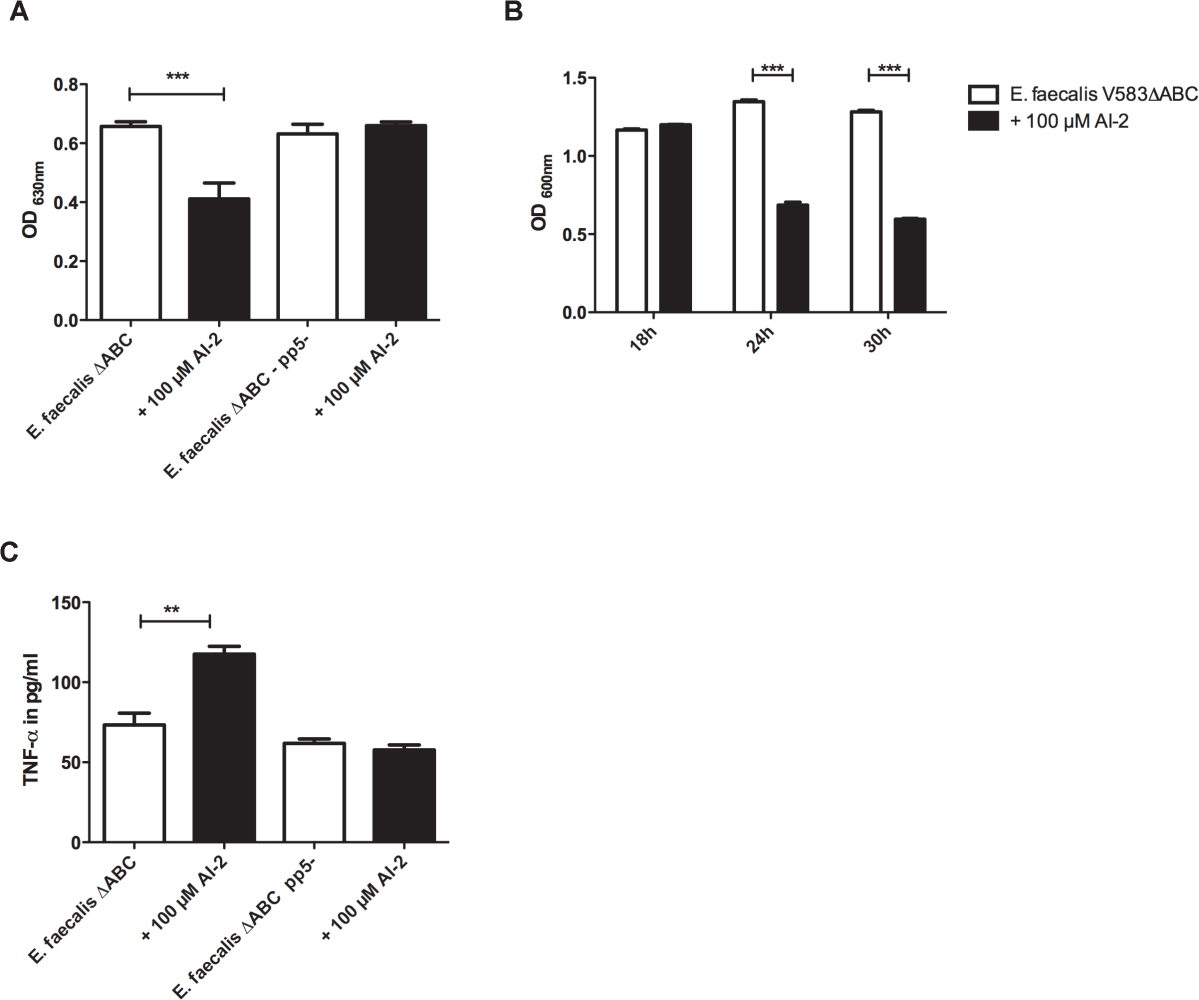
Phenotypic characterization of *E. faecalis* V583ΔABC*pp5-* in the absence or presence of 100 μM AI-2:(A) Biofim assay comparing attachment in the *pp5-* muntant in comparison to the wild type (B) Bioflim assay determining the influence of AI-2 on statically grown biofilms (C) TNF-α release by RAW 264.7 macrophages after stimulating cells with AI-2 treated or untreated bacteria was measured to study the influence of prophage 5 on virulence. **(A)** Ability to form biofilms in microtiter plates in the presence of 100 μM AI-2 was measured in *pp5-* mutant and *E. faecalis* V583ΔABC. Staining of the attached bacteria was done with crystal violet and absorbance was measured at 600 nm **(B)** A static biofilm was grown over 18 h and after 24 and 30 h, AI-2 was added which resulted in a decrease in both strains. **(C)** TNF-α release by RAW 264.7 macrophages stimulated with supernatants of V583ΔABC and the *pp5-* mutant was compared in the presence and absence of AI-2. Statistical analysis of all three experiments was done by ANOVA (p<0.001) with Dunnett post test. ** indicates p<0.01, *** p<0.001. Error bars represent standard error of the mean.

To further investigate the effects of AI-2 on transcription, we performed RNA-seq after exposing bacteria to 100 μM AI-2. Supplementation of bacterial culture with AI-2 resulted in up-regulation of 28 genes (up to log_2_ FC 1.21) (S3 Table). All significantly up-regulated genes are located in a single cluster on the bacterial chromosome (EF2085-EF2113) and belong to prophage 5 (*pp5*)—one of seven prophages present in the genome of *E. faecalis* V583ΔABC. To determine whether the decrease of biofilm formation after addition of 100 μM AI-2 is caused by the up-regulation and release of prophage 5, a mutant in *E. faecalis* V583ΔABC lacking prophage 5 (*pp5-*) [3] was tested. In comparison to wild type, the *pp5-* mutant produced biofilm at the same rate regardless of the application of AI-2. (Fig. 2A), indicating that up-regulation of *pp5* is involved in dispersal of biofilms (i.e. EF2086–2087 and EF20093). To study whether AI-2 inhibits biofilm formation or leads to the dispersal of an already formed biofilm, 100 μM AI-2 was added to biofilms statically grown over 18 hours. As demonstrated in Fig. 2B, a significant decrease in biofilm was observed after 24 and 30 hours. To be able to distinguish between biomass and viable cells in biofilms, bacterial growth was also determined (S5 Fig.). While biofilm and bacterial cells increased between 18 and 24 hours, bacterial survival started to decrease after a 30 hours. Nevertheless, the total number of viable bacteria cells decreased significantly after addition of 100 μM AI-2.

To test if up-regulation and release of prophage 5 is associated with host damage and inflammation, we measured cytokine production in response to AI-2-mediated phage release.

The activation of macrophages by bacteria grown with 100 μM of AI-2 was tested and increased TNF-α production by macrophages was observed only in the wild type but not in the *pp5-* mutant (Fig. 2C). These data support the hypothesis that prophage 5 carries specific virulence factors that mediate inflammation in the wild type but not in the *pp5-* mutant, as measured by cytokine production.

**Fig 3:**
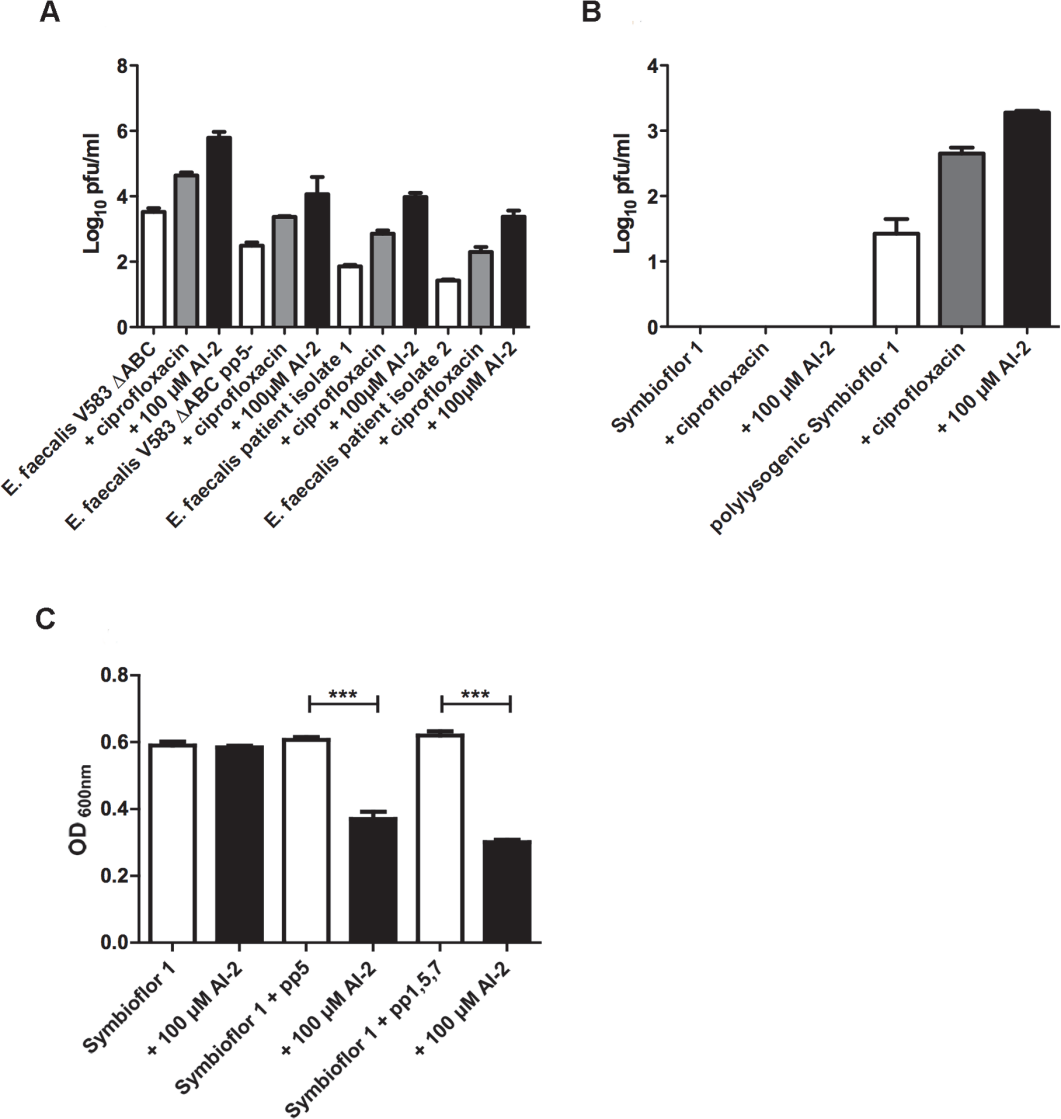
Determination of lytic activity in culture supernatants of *E. faecalis* V583ΔABC,*E. faecalis*V583ΔABC pp5- and two clinical isolates of *E. faecalis* (A). Plaque-forming units of probiotic recipient strain Symbioflor 1 before and after transduction with V583ΔABC phages (B). Comparison of Symbioflor 1 and polylysogenic Symbioflor 1 regarding biofilm formation after induction with 100 μM AI-2 (C). **(A)** Plaque-forming units (pfu) of E. faecalis V583ΔABC *pp5-* and two clinical isolates were grown in medium without supplements, with ciprofloxacin, or with 100 μM AI-2 were determined and compared **(B)** Supernatants of *E. faecalis* V583ΔABC, grown in the presence of AI-2 or ciprofloxacin, were used to transduce probiotic strain *E. faecalis* Symbioflor 1, and plaque-forming units of the transduced and untransduced strain was determined. **(C)** A microtiter plate biofilm assay was done with staining of the attached bacteria by crystal violet. Biofilm formation was measured in absence or presence of 100 μM AI-2. Absorbance was measured at 600 nm. Statistical analysis for biofilm analysis was done by ANOVA (p<0.001) with Dunnett post test. *** indicatesp<0.001. Error bars represent standard error of the mean.

The genome of the wild type *E. faecalis* V583ΔABC contains seven prophages, and Matos *et al*. showed previously that ciprofloxacin and mitomycin induces the release of prophages 1, 5 and 7. We therefore investigated whether AI-2 leads not only to the expression of genes encoding prophage 5 but also to increased phage release and to the release of prophages other than pp5. To analyze the lytic activity of prophages in *E. faecalis* V583*Δ*ABC and the two clinical isolates in comparison to the *pp5-* mutant, bacterial strains were grown for 12 h in the absence or presence of 100 μM AI-2 or ciprofloxacin, and plaque-forming units (pfu) were counted. Induction with AI-2 resulted in significantly higher numbers of plaques (pfu) compared to ciprofloxacin in all tested *E. faecalis* wild type strains. The *pp5-* mutant also produces plaques although the total amount of plaques after induction with AI-2 or ciprofloxacin is lower compared to the number of plaques produced by the wild type *E. faecalis strains* (Fig. 3A). This observation indicates that AI-2 is a strong inducer of prophages in *E. faecalis*, however not the only one and not only for prophage 5.

**Fig 4:**
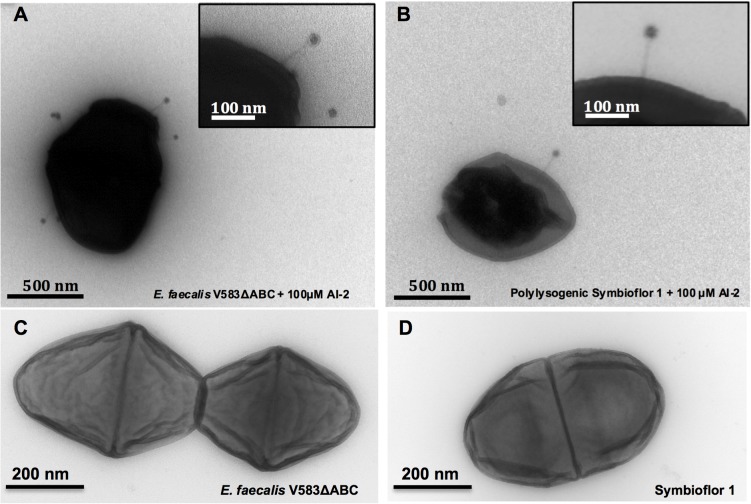
Transmission Electron Micrographs of *E. faecalis* V583ΔABC and Symbioflor 1 surface-associated phages after induction with 100 μM AI-2 and controls. **(A)** *E. faecalis* V583ΔABC induced with 100 μM AI-2 (scale bar, 500 nm), **(B)** *E. faecalis* Symbioflor 1 transfected with phages from *E. faecalis*V583ΔABC and induced with 100 μM AI-2 (scale bar 500 nm). Insets represent higher magnifications of phages at the surface of the cell (scale bar 100 nm). **(C)** *E. faecalis* V583ΔABC without supplementation (scale bar 200 nm) and **(D)** Symbioflor1 without supplementation of AI-2 (scale bar 200 nm).

Being a gut commensal present in the flora of healthy individuals, virtually avirulent enterococci have been used as probiotics to benefit health. Therefore we investigated whether a probiotic *E. faecalis* strain Symbioflor 1 [19] can be transduced by phages released from *E. faecalis* V583ΔABC. As expected, induction of prophages in *E. faecalis* Symbioflor 1 with 100 μM AI-2 did not lead to plaque formation, confirming that Symbioflor 1 does not contain lytic prophages [19] (Fig. 3B, first three columns). Next, Symbioflor 1 was exposed to phage-containing culture supernatants from *E. faecalis* V583ΔABC in which phages were released by AI-2. To measure the efficacy of transduction, transfected Symbioflor 1 was induced by AI-2 or ciprofloxacin. Plaques were visible in the transfected Symbioflor 1 (Fig. 3B, last three columns) indicating that this strain is infected by phages from pathogenic *E. faecalis* V583ΔABC and that prophages are induced better by AI-2 compared to ciprofloxacin. Transmission electron microscopy (TEM) confirmed release of phage particles upon addition of 100 μM AI-2 in *E. faecalis* V583ΔABC and polylysogenic Symbioflor 1 (Fig. 4).

To confirm the presence of prophages in the genome of Symbioflor 1 we used PCR with primers flanking the *att* sites, combined with unique internal primers for each prophage. Thereby, we could confirm that the polylysogenic transfected *E. faecalis* Symbioflor 1 harbored prophages 1, 5 and 7 (Table 1). As described previously, all Symbioflor 1 strains contain also pp2 as part of the core genome [19].

**Table 1:** PCR results validating the presence or absence of phage sequences in the original *E. faecalis* Symbioflor 1, polylysogenic Symbioflor 1, and bacterial isolates from animals infected with polylysogenic Symbioflor 1.

isolate	pp1	pp2	pp3	pp4	pp5	pp6	pp7
inoculum	+	+	-	-	+	-	+
M1; Gr.2 liv	-	+	-	-	+	-	-
M2; Gr.2 liv	+	+	-	-	+	-	+
M3; Gr.2 liv	+	+	-	-	+	-	+
M4; Gr.2 liv	-	+	-	-	+	-	-
M5; Gr.2 liv	-	+	-	-	+	-	-
M6; Gr.2 liv	-	+	-	-	-	-	-
M7; Gr.2 liv	-	+	-	-	-	-	-
M8; Gr.2 liv	-	+	-	-	+	-	-

Pp1 to 7 represent the seven prophages from *E. faecalis* V583 ΔABC, + indicates that sequences of that prophage were found in the respective isolate by PCR. The results confirm that the inoculum strain *E. faecalis* Symbioflor 1 was stably transduced with prophages pp1, pp5 and pp7, while bacteria isolated from animals contained pp1, pp5, and pp7, only pp5, or only pp1 and pp7. All Symbioflor 1 strains contain pp2 (part of the core genome). Primers used are shown in S2 Table.

To study the influence of prophage of polylysogenic Symbioflor 1 on dispersal of biofilms, a biofilm assay was performed after supplementation with AI-2. Addition of 100 μM AI-2 resulted in a significantly reduced biofilm in both transduced strains (Symbioflor 1 + pp5 and Symbioflor 1 + pp1, 5 and 7) compared to the untransduced wild type strain (Fig. 3C). Determining the total number of viable cells in the biofilm showed a decrease in biofilm and in viable bacterial cells after the addition of 100 μM AI-2 (S5 Fig.).

**Fig 5:**
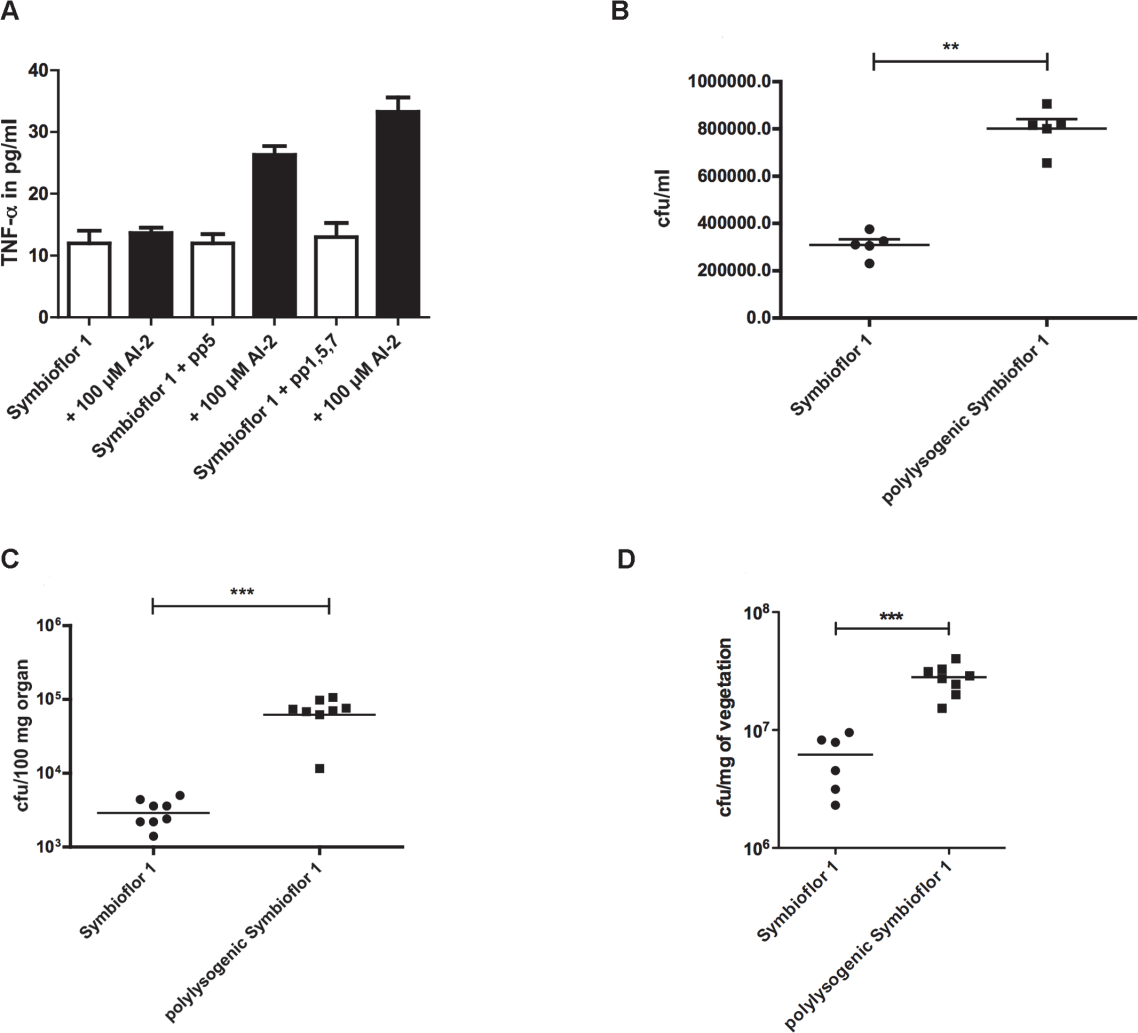
Influence of phages on virulence of *E. faecalis* Symbioflor 1: Virulence of polylysogenic Symbioflor 1 was compared to Symbioflor1 *in vitro*, a TNF-α assay (A) and an adherence assay to CaCo cells (B) was done. Both strains were also compared *in vivo* in a mouse bacteremia model (C) and a rat endocarditis model (D). **(A)** Inflammatory response was determined measuring TNF-α release of RAW 264.7 macrophages after stimulation with polylysogenic Symbioflor 1 with 100 μM AI-2. Statistical analysis was done vy ANOVA (p<0.001) with Dunnett post test and *** indicates p<0.001. Error bars represent standard error of the mean **(B)** Adhesion of probiotic *E. faecalis* Symbioflor 1 and polylysogenic Symbioflor 1 to colon epithelial cells (CaCo cells) was compared. Statistical analysis was done by ANOVA (p<0.01) and ** indicates p<0.001. **(C)** Mouse bacteremia model with 9.9 x 10^7^cfu/mouse (Symbioflor 1) and 8 x 10^7^ cfu/mouse (polylysogenic Symbioflor) injected i.v.; cfu in the liver was assessed after 24h. Statistical analysis was determined by Mann Whitney and *** indicates p<0.001. **(D)** A rat endocarditis model shows increased colony-forming units in vegetations formed by polylysogenic Symbioflor 1. Inoculation of bacteria followed 48 h after catheter placement via injection into the tail vein. Bacterial counts in the vegetations (cfu per gram vegetation) was assessed. Statistical significance was determined by Mann-Whitney U test and *** means p<0.001.

To test whether infection of bacteria with phages has an influence on phenotype and especially on virulence, we compared the polylysogenic *E. faecalis* Symbioflor 1 (containing pp1, pp2, pp5, and pp7) to the original uninfected probiotic strain (containing only pp2). The inflammatory response of macrophages was tested by infecting RAW 264.7 macrophages with transduced and untransduced Symbioflor 1 grown in the presence or absence of AI-2. Both transduced strains showed a stronger TNF-α release compared to untransduced Symbioflor 1 (Fig. 5A). Furthermore, adherence to colonic epithelium (Caco2 cells) was quantified *in vitro* confirming that polylysogenic Symbioflor 1 showed an approximately two-fold higher binding to Caco2 cells compared to the original probiotic strain (Fig. 5B). These findings underline the impact of phages on virulence and colonization.

A mouse bacteremia model was used to compare the virulence of polylysogenic with the un-transduced *E. faecalis* Symbioflor 1. To confirm the presence of prophages in *E. faecalis* Symbioflor 1 after transduction, PCR was performed before using this strain as inoculum in the animal model. In addition, PCR was also performed after isolating colonies from infected animals to confirm the presence of prophages in transfected *E. faecalis* Symbioflor 1 (Table 1). Primers were used as described above and sequences are listed in supplementary S2 Table. Liver colony counts were about 10 times higher in animals infected with polylysogenic *E. faecalis* Symbioflor 1, as compared to the original untransduced Symbioflor 1. In addition, all animals infected with the polylysogenic *E. faecalis* Symbioflor 1 strain appeared sicker (i.e., ruffled fur, limited movement) at the end of the experiment (Fig. 5C). The virulence of *E. faecalis* Symbioflor 1 was compared to *E. faecalis* Symbioflor 1 transduced either with prophage 5 (S6 Fig.). *E. faecalis* Symbioflor 1 + pp5 showed significantly higher colony forming units in the kidney compared to the probiotic wild type, confirming the importance of prophage 5 in virulence. Similar results were obtained with a rat endocarditis model showing increased colony-forming units in vegetations formed by polylysogenic Symbioflor 1 (Fig. 5D). These results provide evidence that an apparently avirulent probiotic strain can become more pathogenic through the uptake of virulence genes transmitted by phages.

## Discussion

Phage-mediated transfer of virulence and antibiotic resistance genes has been known as an important mechanism for gene transfer among bacterial strains and species [20]. Here we show that enterococci use AI-2 as a communication molecule and propose a novel mechanism of AI-2-dependent phage induction regulating biofilm formation in *E. faecalis*. We identified several genes regulated by AI-2 and found that 28 genes significantly up-regulated by high AI-2 concentrations belong to one of the seven prophages of *E. faecalis* V583ΔABC.

It has been shown previously that AI-2 is a signal molecule as well as a detoxification product of the activated methyl cycle [21]. Cell-to-cell signaling involves more than simply the presence of a toxic or nutritional molecule per se [22]. Certain toxic metabolites can provoke a stress response once a critical concentration has been reached. Therefore, a cell-to cell signal molecule needs to be defined with caution. Nevertheless, we believe that *E. faecalis* uses AI-2 as a cell-to cell signal molecule arising from a certain population density to infect neighboring bacteria and to adapt to its environment. However, there are experimental data for *S. aureus* that AI-2 may be more important as a metabolite as opposed to a Quorum sensing signal [23].

The impact of AI-2 on biofilm formation was shown previously by Shao and colleagues and they also proposed that AI-2 may play a role in the regulation of a number of important metabolic pathways [24]. None of the genes identified by these authors were found in our transcriptome analysis. However, this discrepancy is explained by the different experimental settings: the results of Shao *et al*. identified genes up-regulated at 2 h, while our RNA-seq data (taken at 6 h) show a different set of genes that were up-regulated at the later time point. Moreover, the concentration of AI-2 used by Shao and colleagues was between 0.2–20 μM and was therefore much lower compared to the concentrations used in the present study (100 μM AI-2). Influence of AI-2 concentration on biofilm formation was reported previously by Rickard [10] and Cuadra-Saenz [25] in *Streptococcus oralis* and *S. gordonii*, showing that high AI-2 concentrations interfere with the communication signals bacteria need to establish complex biofilm architectures.

Several studies have characterized ubiquitous phages in enterococci and have elucidated important aspects of the underlying pathogenicity, the role of prophage dynamics, and provided evidence for their impact on evolution towards pathogenicity [3,26,27]. Matos et al. showed that six of seven prophages (i.e. pp1, pp3, pp4, pp5, pp6, and pp7) are inducible and four of them form infectious virions regulated by a network that was described as the first "enterococcal phage-related chromosomal island" [3]. They also demonstrated that pp1 and pp7 only transfer together [3].

We observed a decrease in biofilm when 100 μM AI-2 was added and concluded that this represents dispersal of an already formed biofilm. This hypothesis is concordant with the electron micrographs and the up-regulation of phage genes that are involved in lysis (i.e. EF2086—an endolysin, and EF2087—a holin). However, additional experiments are needed to elucidate the underlying mechanism of dispersal of biofilm through phage release. Further studies should also determine whether an endogenous gradual increase of AI-2 up to 100 μM leads to similar effects (e.g. phage release and/or pp5 induction) compared to the sudden increase through the addition of 100 μM exogenous AI-2.

Based on our results we hypothesize that AI-2 plays an important role in expression and distribution of virulence factors. With increasing bacterial density, bacteria from biofilms must disperse, migrate across epithelial linings, and disseminate by overcoming the host’s antimicrobial defense systems. These adaptive mechanisms require a set of virulence factors, which are induced by increasing bacterial density and consequently higher concentrations of AI-2 in the microenvironment. To confirm this hypothesis, a LuxS mutant would be helpful. However, while our attempts to create such a mutant were futile, others were able to create a LuxS mutant in *E. faecalis* but were not able to complement this mutant, probably due to the essential role of LuxS in detoxification or due to second site suppression (Lynn Hancock, personal communication).

Phage release in *E. faecalis* is induced either by antibiotics (such as ciprofloxacin) [3,26] or by higher amounts of AI-2. Both situations may be encountered in the gastro-intestinal tract of hospitalized neutropenic patients, who often receive quinolone antibiotics for prophylaxis, leading to a selective enrichment of enterococci in the intestinal flora. As a consequence, released phages become much more likely to infect neighboring bacteria, leading to an increase in virulence, as demonstrated in our mouse bacteremia model. Via stool analysis of patients on quinolone antibiotics, an expansion of the phage metagenome has been reported recently by Modi and colleagues [2]. This mechanism greatly increases the spread of virulence and resistance genes through phages and helps the bacteria to adapt to the appropriate stage of infection and the ecological environment [27]. A particularly worrying observation is that previously phage-free "apathogenic" strains (like the probiotic Symbioflor 1) may be transduced by the phages of more virulent strains (such as *E. faecalis* V583 ΔABC), leading to a rapid spread of virulence factors among enterococci in the gastrointestinal tract of hospitalized patients.

## Materials and Methods

### Bacterial strains and mutants

Bacterial strains and plasmids used in the present study are shown in S1 Table. Enterococci were grown without agitation at 37°C in tryptic soy broth (TSB; Becton Dickinson). Growth was monitored by measuring optical density at 630 nm (OD_630nm_).

### Biofilm

Biofilm formation of *E. faecalis* V583ΔABC,*E. faecalis* ΔABC*pp5-, E. faecalis* 12030, two clinical isolates and Symbioflor1 (including transduced strains with prophage 5 and polylysogenic Symbioflor 1) was measured as described by Theilacker *et al*. in the presence of AI-2 [28]. In brief, bacteria were grown in TSB containing 1% (w/v) of glucose for 18 h at 37°C. Polystyrene 96-well tissue-culture plates (Greiner bio-one) were filled with 180 μl of fresh TSB (with 1% (w/v) glucose), and 20 μl of the overnight culture was added to each well. To test the influence of AI-2 (OMM Scientific Inc.) on production of biofilms, AI-2 was supplemented to provide final concentrations 100 μM. The plates were incubated for 12 h at 37°C and measured in an ELISA reader (Synergy H1 Hybrid Reader, Bio-Tek) at an optical density of 630 nm, to assure equal growth in all wells. Culture medium was discarded, and wells were carefully washed 3 times with 200 μL of PBS (Biochrom). Plates were dried for 40 minutes at 50°C and stained afterwards with crystal violet (Sigma) for 2 minutes. Excess stain was removed by rinsing the plates under tap water and drying for 10 minutes at 50°C. The absorbance at 600 nm was determined, and each assay was performed in triplicate and repeated three times. The optical density was analyzed by ANOVA with Dunnett post test. For the biofilm experiments, we always measure the final OD after overnight growth before measuring the biofilm formation (biofilm = OD_600nm*0,5_/OD_630nm_) to assure equal growth in all wells. These OD values of the planctonic culture of *E. faecalis* and Symbioflor strains were measured before staining the biofilm and no statistically significant difference was observed between these ODs.

### Determination of bacterial survival in biofilms

To study the number of viable bacterial cells in biofilms, bacteria were grown in Petri dishes and collected after 1, 6, 24 and 30 hours by scraping attached bacteria off the plates. The total number of bacteria was determined using a Thoma cell counting chamber and, in parallel, bacteria were plated on TSA plates to quantify the number of colony forming units. Finally, bacterial cell survival was calculated as follows: cfu / total cell number * 100 [29].

### FRET assay for AI-2 quantification

The concentration of AI-2 in filter-sterilized supernatants of *E. faecalis* V583ΔABC and *E. faecalis* V583ΔABCΔ*pp5-* were determined by a FRET-based AI-2 assay [30,31]. Briefly, the reporter protein consisting of CFP, LuxP, and YFP (CLPY) was produced in an AI-2 *E. coli* strain, purified by Ni-NTA affinity chromatography and used for the assay in a final concentration of 0.025 mg/ml in reaction buffer (25 mM sodium phosphate buffer pH 8.0, 35 mM sodium chloride and 1 mM boric acid). AI-2 (OMM scientific, Texas) in solution or supernatants (when necessary diluted with medium) was added, and the decrease in fluorescence resonance energy transfer induced by ligand binding was determined. The fluorescence emission measured at 540 nm and 485 nm upon excitation at 430 nm gives the FRET ratio (YFP/CFP). A calibration curve for AI-2 (1–60 μM) was used to determine the concentration of AI-2 in the supernatants [32,33]. All experiments were done in triplicate at different time points.

### RNA-sequencing

Strain *E. faecalis* V583 was inoculated to an OD_630nm_ of 0.1 from a freshly prepared overnight culture and grown for 6 h in TSB, with one culture without AI-2 and one with 100 μM AI-2 (OMM Scientific Inc). After 6 hours RNA was isolated (Qiagen) and analyzed for gene expression profiles. RNA-seq sequencing libraries were prepared from 1 μg of total *E. faecalis* V583 RNA. First, ribosomal RNA species were removed using the RiboZero rRNA Removal Kit (Epicenter). Subsequently, sequencing libraries were prepared with the TruSeq RNA Sample Preparation Kit v2 (Illumina) according to the manufacturer’s instructions. The libraries were tagged with a 6-nt-long barcode sequence in the sequencing adapter to allow sequencing of pooled samples. A pool of 9 libraries was loaded onto two lanes of an Illumina GAIIx flow cell, and 50 nt were sequenced in single-read mode. On average 8.5 ± 1.9 Mio reads were generated per sample. De-multiplexing and generation of fastq files were done with GERALD/ELANDv2 (Illumina). Alignment of reads against the *E. faecalis* V583 genome was performed with bowtie (parameters: -n 3 -l 20 -k 3 —best —strata) [34]. On average 8.1 ± 1.8 Mio reads mapped to non-rRNA locations in the genome. The coordinate-sorted bam files of all nine samples were used to count reads for each gene using bedtools v2.16.2. Here the Refseq genes were retrieved from the UCSC microbial genome browser (http://microbes.ucsc.edu). Regions in which genes overlap were filtered out, since the library preparation protocol was not strand conserving. This annotation file was used to count reads for each feature in each bam file. Reads with fewer than 5 counts in any sample were removed from the data set (41/5349), and differentially expressed mRNAs were calculated using the Bioconductor package ‘edgeR’ [35] using the generalized liner model approach. RNAs with a false discovery rate of less than 0.05 were called differentially expressed.

### Extraction of chromosomal DNA and PCR conditions

Chromosomal DNA was extracted using the MasterPure Gram Positive DNA Purification Kit according to the manufacturer's instructions (Epicentre). For each isolate, the DNA concentration was estimated using a NanoDrop spectrophotometer (ND-2000, peqlab), and DNA integrity was verified using a 0.8% agarose gel. A primer pair EFS1_2450, being unique for Symbioflor, was used to confirm the identification of *E. faecalis* Symbioflor 1. The presence of the prophages pp1-pp7 in the genome of Symbioflor was assessed by differential PCR.

A set of 3 primers was used: Primer set A is flanking the *att* sites for the specific prophage and binds in the genome of Symbioflor 1. A fragment amplification using this primer set indicates no integration of corresponding phage. Primer set B consists of a forward primer binding ca. 200 bp upstream of the *att* site at the Symbioflor 1 genome and a reverse primer binding within the first 500bp of the prophage sequence. Primer set C consists of a primer pair, binding a unique gene from each single prophage. Positive PCR results using primer sets B and C confirms the phage integration into Symbioflor 1 genome. As a control we included a primer pair amplifying a unique gene present in the genome of *E. faecalis* Symbioflor 1 (e.g. EFS1_2450). Primersets A, B and C are listed in S2 Table and amplified by using TopTaq DNA Polymerase (QIAGEN) using conditions as recommended by the manufacturer in a thermocycler peqSTAR 2X (peqlab).

### Induction of TNF-alpha

A TNF-α assay was performed to assess the impact of the AI-2 concentration on the expression of bacterial compounds recognized by the innate immune system. Bacterial cells were grown at 37°C in TSB medium in the presence or absence of AI-2 (100 μM) to an optical density OD_630nm_ of 0.7. Culture supernatants were collected and filter sterilized by filtration using a 0.22-μm-pore-size filter (Millipore). RAW 264.7 cells were seeded in 48-well plates to a density of 1 × 10^5^ cells/well and incubated with supernatants for 16 h. Supernatants of RAW cells were collected and analyzed by commercial ELISA according to the recommendations of the manufacturer (eBioscience). Lipopolysaccharides from *E. coli* (Sigma) were used as positive controls. The ELISA was performed in triplicate and repeated three times. Statistical analysis of variance was performed with ANOVA.

### Phage lytic activity in culture supernatants

To analyze the lytic activity of phage particles in *E. faecalis* V583*Δ*ABC, *pp5-*, Symbioflor 1 and the two clinical isolates strains were grown for 12 h overnight in the absence or presence of 100 μM AI-2 or ciprofloxacin (4μg/ml; Fresinius KABI). From these cultures, several aliquots and dilutions were taken (100 μl total volumne), mixed with 5 ml TSB soft agar and poured onto freshly prepared TSA plates. For preparation of soft agar, 200 ml of fresh TSB medium was supplemented by 1.4 g agar, melted and subsequently cooled to 37°C. Plates were incubated at 37°C for 18 h and plaque-forming units (pfu) were counted. Plaques titers were calculated as follows: pfu/ml = (number plaques/plate)*(1/ml plated)*dilution factor. All experiments were done in triplicate, and statistical significance was analyzed with ANOVA.

### Transduction of *E. faecalis* Symbioflor 1

Cell-free supernatants of a 12 h statically grown culture of *E. faecalis* V583*Δ*ABC, grown in the absence or presence of 100 μM AI-2, were collected, centrifuged, and filter-sterilized using a 0.22-μm-pore-size filter (Millipore). Following the procedures described by Matos *et al.*, ciprofloxacin (Fresenius KABI) was added to a final concentration of 4 μg/ml. In parallel, *E. faecalis* Symbioflor 1 was cultured overnight and freshly inoculated to achieve a total number of 10^5^ cells. A total of 10^5^ *E. faecalis* Symbioflor 1 cells were incubated with the phage-containing supernatant of *E. faecalis* V583*Δ*ABC. The ratio between phage and bacteria was chosen to be 1:1 and the phage-bacteria mix was incubated in TSB for 2 h at 37°. After incubation, 100 μl of this suspension was mixed with 5 ml softagar and applied to TSA plates; several dilutions for exact determination were plated. For preparation of soft agar, 200 ml of fresh TSB medium was supplemented by 1.4 g agar (BD), melted and subsequently cooled to 37°C. Plates were incubated at 37°C for 18 h and plaque-forming units (pfu) were counted. Plaques were calculated as follows: pfu/ml = (number plaques/plate)*(1/ml plated)*dilution factor/10^5^ *E. faecalis* Symbioflor 1 cells. To verify a stable transduction, single plaques were picked, grown overnight, and chromosomal DNA was extracted. Confirmation of the presence or absence of the seven prophages was done by PCR as described above/below. Successfully transduced strains were used for the animal model and lytic activity of transduced Symbioflor 1 strain was determined as described above. All experiments were done in triplicate, and statistical significance was analyzed with ANOVA.

### Adhesion to eukaryotic cells

Adhesion of bacteria to Caco-2 cells was tested similarly to the method described by Rigottier-Gois *et al*. for Caco-2 cells [36]. Caco-2 cells were incubated with *E. faecalis* Symbioflor 1 and transduced Symbioflor. Caco-2 cells were cultivated in 24-well plates to a density of 1 × 10^5^ cells/well for 13–15 days. Bacteria were grown to mid-log phase at 37°C without agitation in tryptic soy broth; Caco-2 cells were incubated with bacteria for 2 h at a multiplicity of infection (MOI) of 100. After infection of the monolayer, epithelial cells were washed with phosphate saline buffer (PBS, Biochrom AG) and lysed with 0.25% Triton-X (Sigma) at 37°C for 15 minutes. Adherent bacteria were enumerated by quantitative bacterial counts.

### Animal experiments

The virulence of probiotic *E. faecalis* Symbioflor 1 and polylysogenic *E. faecalis* Symbioflor 1 was compared in a mouse bacteremia model as described previously [16]. Eight female BALB/c mice 6–8 weeks old (Charles River Laboratories Germany GmbH) were infected by i.v. injection of *E. faecalis* Symbioflor 1 (9.9 x 10^7^ cfu) or transduced *E. faecalis* Symbioflor 1 (8.0 x 10^7^ cfu) via the tail vein. Twenty-four hours after infection, mice were sacrificed and livers/kidneys were aseptically removed, weighted and homogenized. Bacterial counts were enumerated by serial dilutions on TSA plates after overnight incubation. Statistical significance was assessed by Mann-Whitney test.

Female Wistar rats (Charles River Laboratories Germany GmbH), weighing 200 to 300 g were used in model as described elsewhere [37]. The animals were anesthetized by subcutaneous application of 5.75% ketamine and 0.2% xylazine. Nonbacterial thrombotic endocarditis was caused by insertion of a small plastic catheter (polyethylene tubing; Intramedic PE 10) via the right carotid artery. The artery was accessed by cutting the neck laterally on the right side. The carotid artery could be easily exposed and ligated distally. The polyethylene catheter was introduced via a small incision of the vessel and advanced until a slight resistance indicated passage through the aortic valve. The catheter was advanced through the aortic valve into the left ventricle. Proper placement was ensured via invasive pressure measurement through the catheter’s lumen. To minimize confounding factors we focused on standardization of the catheter insertion and its positioning. Preliminary experiments without secondary bacterial colonization showed that pressure monitoring and therewith objectification of catheter positioning could minimize overly traumatic injury (which may lead to pronounced thrombotic lesions) and ensured constant lesions. The catheter was secured in place and distally ligated. A simple running suture was used for wound closure. Postoperatively the rats were returned to their cage and monitored closely.

Inoculation of bacteria followed 48 h after catheter placement via injection into the tail vein. Rats were assigned to two groups, 6 receiving the wild type strain Symbioflor 1, and 8 being challenged with transduced Symbioflor 1. Animals were sacrificed on postoperative day 6 and the correct placement of the catheter was verified. The extent of native valve endocarditis was assessed and graded macroscopically, and subsequently valve vegetations were removed aseptically. After weighing of the vegetations, 500 μL TSB was added per sample and the vegetations were homogenized.

The primary evaluation criterion was the bacterial count in the vegetation (cfu per gram and per ml, respectively). The mean and standard deviation was calculated for each group. Statistical significance was determined by Mann-Whitney U test. The appropriate sample size was calculated using Mead´s resource equation.

### Scanning electron microscopy

Biofilms grown on poly-L-lysine-coated glass slides (BD biosciences) were mock treated or treated with 100 μM AI-2 in growth medium in 12-well-plates and carefully washed with 2 x 1 ml phosphate-buffered saline (PBS) (Biochrom) to remove medium and fixed for 1 h with 2% (v/v) glutaraldehyde (Sigma) in PBS at room temperature. Samples were washed twice with PBS to remove excess fixative and subsequently serially dehydrated by consecutive incubations in 1 ml of 25% (v/v) and 50% (v/v) ethanol-PBS, 75% (v/v) and 90% (v/v) ethanol-H_2_O, and 100% ethanol (2x), followed by 50% ethanol-hexamethyldisilazane (HMDS) and 100% HMDS (Sigma). The glass slides were removed from the 100% HMDS and air-dried overnight at room temperature. After overnight evaporation of HMDS, samples were placed on specimen mounts (Ted Pella Inc.) and biofilms coated with 80% Pt-20% Pd to 4 nm using a Cressington 208HR sputter coater at 40 mA prior to examination with a Phenom Table-top scanning electron microscope.

### Transmission electron microscopy

Transmission electron microscopy was performed as described previously with some modifications [38]. *E. faecalis* cells mock treated or induced with 100 μM AI-2 for 6 hours were resuspended in 1% (v/v) glutaraldehyde (Sigma). Copper grids (mesh Formvar-carbon coated) were incubated for 30 mins with carbon side on a drop of *E. faecalis* cells +/- AI-2. Grids were washed 3 times for 5 mins with drops of 0.02 M glycine in PBS followed by 5 consecutive washes in ultra-pure H_2_O. Bacteria and phages were stained by incubation of the grids for 5 mins on drops containing 1.8% (w/v) methylcellulose (25 centipoises; Sigma-Aldrich) and 0.4% (w/v) uranyl acetate (pH = 4) and subsequently air dried for 10 min. Grids were examined using a Jeol 1010 transmission electron microscope (Jeol Europe, The Netherlands).

### Ethical statement

All animal experiments were performed in compliance with the German animal protection law (TierSchG). The animals were housed and handled in accordance with good animal practice as defined by FELASA and the national animal welfare body GV-SOLAS. The animal welfare committees of the University of Freiburg (Regierungspraesidium Freiburg Az 35/9185.81/G-12/070 and Az 35/9185.81/G-07/72) approved all animal experiments. The institutional review board of the University of Freiburg approved the study protocol.

### Statistical analysis

All statistical testing was done using GraphPad PRISM with ANOVA and Dunnett, or Tukey post tests as indicated. Two-group comparisons were done by unpaired t-test. Animal experiments were analysed by Mann Whitney test.

## Supporting Information

S1 Table Strains and plasmids used in this study. (DOC)

S2 Table Primers that were used in this study. (DOCX)

S3 Table Results from RNAseq. (DOCX)

S1 Fig Determination of the AI-2 concentration produced by *E. faecalis* V583ΔABC*pp5-*. (DOCX)

S2 Fig Determination of the AI-2 concentration in the supernatants of *E. faecalis* V583ΔABC after supplementation with 100 μM AI-2. (DOCX)

S3 Fig Scanning microscopy. Panel A shows biofilm formation without supplementation of AI-2. Panel B shows biofilm formation after supplementation with AI-2 of *E. faecalis* V583 ΔABC.
(DOCX)

S4 Fig Growth curve of *E. faecalis* V583 ΔABC in absence or presence of 100 μM AI-2. (DOCX)

S5 Fig Bacterial survival of *E. faecalis* V583ΔABC, Symbioflor 1, Symbioflor transduced with prophage5 and polylysogenic Symbioflor. Panel A shows viable cell numbers during biofilm formation of *E. faecalis* V583ΔABC. Panel B demonstrates viable number of Symbioflor 1 and transduced Symbioflor 1 during biofilm formation.
(DOCX)

S6 Fig Mouse bacteremia model comparing Symbioflor wild-type and transduced Symbioflor strains. (DOCX)
