# Supplementary material for: Phage-mediated Dispersal of Biofilm and Distribution of Bacterial Virulence Genes Is Induced by Quorum Sensing
Source: PLoS Pathog. 2015 Feb 23;11(2):e1004653. doi: 10.1371/journal.ppat.1004653 (PMC4338201; doi:10.1371/journal.ppat.1004653)
Supplement: S1 Table — (DOC) [file ppat.1004653.s001.doc]

**Table S**1: Strains and plasmids used in this study

| **species** | **description** | **strains** | **Presence of prophages** | **ref.** |
| --- | --- | --- | --- | --- |
| *E. faecalis* | V583∆ABC | VE14089 - derivative of *E. faecalis* V583  cured of plasmids A, B. and C | prophage 1, 2, 3, 4, 5, 6, 7 | 33 |
| *E. faecalis* | V583ΔABC*pp5-* | VE14285 (V583∆ABC cured from prophage 5) | prophage 1, 2, 3, 4, 6, 7 | 3 |
| *E. faecalis* | Symbioflor 1 | Symbioflor 1 Clone DSM 16431 | prophage 2,6 | 19 |
| *E. faecalis* | polylysogenic  Symbioflor | Symbioflor 1 Clone DSM 16431, transduced with phages from V583 | prophage 1, 2, 3, 4, 5, 6, 7 | this study |
| *E. faecalis* | 12030 | 12030 | unknown | 16 |
| *E. faecalis* patient isolate 1 | clinical isolate 1 | Provided by bacteriology Lab; Dr. von Hauner spital, LMU, Munich | unknown |  |
| *E. faecalis* patient isolate 2 | clinical isolate 2 | Provided by bacteriology Lab; Dr. von Hauner spital, LMU, Munich | unknown |  |
