# Supplementary material for: Phage-mediated Dispersal of Biofilm and Distribution of Bacterial Virulence Genes Is Induced by Quorum Sensing
Source: PLoS Pathog. 2015 Feb 23;11(2):e1004653. doi: 10.1371/journal.ppat.1004653 (PMC4338201; doi:10.1371/journal.ppat.1004653)
Supplement: S2 Table — (DOCX) [file ppat.1004653.s002.docx]

|  | **PCR primer** | **Sequence** |
| --- | --- | --- |
| 1 | pp1A_FRW | ACAGGTGTTGATATCGTTGATGGTC |
|  | pp1A_REV | GATTCGGATGGAAGCTCGCTTACA |
| 2 | pp1B_REV | CCACAAAAGTTAGCACGCATGTGTT |
| 3 | pp1C_FRW | TGGCGTACAGCCAATAAACGCA |
|  | pp1C_REV | TTCCGCCCAATCAGGAACTACAGA |
| 4 | pp2A_FRW | CGCGTCCAAGGCATCACTATTACA |
|  | pp2A_REV | TCGCCACTAACATCTTCGGTTGTC |
| 5 | pp2B_REV | ACGGCCAAAATCCGATGAACT |
| 6 | pp2C_FRW | AATCAGTCAGCCACGGTCATGAAC |
|  | pp2C_REV | ATTCCTGGAGCCATCACGCTTT |
| 7 | pp3A_FRW | CAAAAGCGTTCGAAGGTACGTTGC |
|  | pp3A_REV | GTCAATCAGCGAAGAACCCAACTC |
| 8 | pp3B_REV | CGACAAGCAGGAGTTCCACTTGAG |
| 9 | pp3C_FRW | CGACTGAAAATTGCGCAGAACGAG |
|  | pp3C_REV | CATCAAGTGGACGCCACTGAATCT |
| 10 | pp4A_FRW | ACGTAAATCACCCGTCTGCTCT |
|  | pp4A_REV | CAACTTCGTTTTGCGCAACAGC |
| 11 | pp4B_REV | TTCGTAGTCGCCTTCTAGGCA |
| 12 | pp4C_FRW | GAACCTTGTTCCCCTGCAATAC |
|  | pp4C_REV | CGACACGTTGCGAAATCACTGAAG |
| 13 | pp5A_FRW | CTTGTGCACGAGATTTGTACGATT |
|  | pp5A_REV | GCCGATGGATAAAACTGCCACTTG |
| 14 | pp5B_REV | GTGACCATAGACAGCTAATTCAG |
| 15 | pp5C_FRW | GCCTCCATTTGCACGTGTAACTCT |
|  | pp5C_REV | CGCATTACCATTTGATTGGACGAGT |
| 16 | pp6A_FRW | CTCAACTGCTTCTCAGTACGGA |
|  | pp6A_REV | GCCCAAAAGGGTAGTCAAATGACA |
| 17 | pp6B_REV | CTGCACCTTAGTGTTAACGG |
| 18 | pp6C_FRW | CACCCCATATCACGAACTGCATTCA |
|  | pp6C_REV | GAAGGCATCGCTGCAGTAGCTAAA |
| 19 | pp7A_FRW | CAGAAGCACCAAGTACGGTTGGAT |
|  | pp7A_REV | TTGGGGCTTAAAGGCGGAAGACAT |
| 20 | pp7B_REV | TACCGCTGACTGGACAAGTTACCT |
| 21 | pp7C_FRW | GCAAACGGCGATACGTTCCATTAG |
|  | pp7C_REV | CGAAATTGCTTGCGGAGATGCAC |
| 22 | EFS1_0029_FRW | TTAGCCCAGTTTCCGCTGAC |
|  | EFS1_0029_REV | AACTGGCCGTCCAATCACAT |
| 23 | EFS1_2443_FRW | ACACCTAAACCGACTAGAATAGC |
|  | EFS1_2443_REV | GGTCCAATCGCTGAAAGAATGC |
| 24 | EFS1_2450_FRW | AAACGTTGCGAACTCTTGGC |
|  | EFS1_2450_REV | TGTTTTTGGCTGGCGTGATG |

**S2 Table 2: Primers that were used in this study**
