# Supplementary material for: Phage-mediated Dispersal of Biofilm and Distribution of Bacterial Virulence Genes Is Induced by Quorum Sensing
Source: PLoS Pathog. 2015 Feb 23;11(2):e1004653. doi: 10.1371/journal.ppat.1004653 (PMC4338201; doi:10.1371/journal.ppat.1004653)
Supplement: S3 Table — (DOCX) [file ppat.1004653.s003.docx]

**Table S3: Results from RNAseq**

| ID | logFC.conc100uM | logCPM | LR | PValue | FDR | geneName |
| --- | --- | --- | --- | --- | --- | --- |
| EF2103 | 1,213443896 | 8,953846346 | 49,59130998 | 1,70E-11 | 5,56E-08 | EF2103 |
| EF2102 | 1,153201146 | 7,354797695 | 43,33495731 | 3,89E-10 | 6,35E-07 | EF2102 |
| EF2104 | 1,09617321 | 10,44367032 | 41,57439568 | 9,38E-10 | 1,02E-06 | EF2104 |
| EF2101 | 1,130133118 | 8,032952772 | 39,53285347 | 2,60E-09 | 2,13E-06 | EF2101 |
| EF2105 | 1,060099163 | 9,823424599 | 38,18200687 | 5,12E-09 | 3,34E-06 | EF2105 |
| EF2100 | 1,003018695 | 8,152153238 | 33,52402852 | 5,25E-08 | 2,86E-05 | EF2100 |
| EF2109 | 1,027127124 | 7,809183283 | 32,84851886 | 7,36E-08 | 3,43E-05 | EF2109 |
| EF2097 | 0,955708384 | 7,441108794 | 32,01154254 | 1,12E-07 | 4,06E-05 | EF2097 |
| EF2098 | 0,937072723 | 8,796574834 | 32,12254343 | 1,06E-07 | 4,06E-05 | EF2098 |
| EF2106 | 0,974398965 | 8,283516769 | 31,13021909 | 1,74E-07 | 5,68E-05 | EF2106 |
| EF2099 | 0,960867994 | 9,978957263 | 30,70673286 | 2,15E-07 | 6,38E-05 | EF2099 |
| EF2107 | 0,90699724 | 8,01643044 | 29,59374345 | 3,75E-07 | 0,000101977 | EF2107 |
| EF2096 | 0,910134596 | 10,38927851 | 26,80940883 | 1,51E-06 | 0,000351695 | EF2096 |
| EF2095 | 0,866336516 | 8,135759389 | 26,910729 | 1,43E-06 | 0,000351695 | EF2095 |
| EF2092 | 0,919714276 | 7,602229216 | 26,64527224 | 1,64E-06 | 0,000356324 | EF2092 |
| EF2108 | 0,862448517 | 8,557590757 | 26,07567848 | 2,18E-06 | 0,000444121 | EF2108 |
| EF2111 | 0,859330378 | 8,547829455 | 24,2921443 | 5,31E-06 | 0,001019676 | EF2111 |
| EF2110 | 0,796058008 | 7,968355339 | 23,52338474 | 7,80E-06 | 0,001414401 | EF2110 |
| EF2093 | 0,832302979 | 9,459217517 | 22,4072936 | 1,36E-05 | 0,002341249 | EF2093 |
| EF2086 | 0,809854319 | 9,044908599 | 22,16785989 | 1,54E-05 | 0,002507053 | EF2086 |
| EF2094 | 0,757225755 | 7,936441808 | 20,02858033 | 4,48E-05 | 0,006958457 | EF2094 |
| EF2091 | 0,737371246 | 6,938644189 | 17,67167297 | 0,000145427 | 0,021582685 | EF2091 |
| EF2112 | 0,738339413 | 8,704256778 | 16,12994866 | 0,000314359 | 0,044625336 | EF2112 |
| EF2089 | 0,650258482 | 7,201595034 | 14,86472333 | 0,000591788 | 0,080507861 | EF2089 |
| EF2128 | 0,7085472 | 7,291469191 | 13,40388205 | 0,001228525 | 0,154865524 | EF2128 |
| EF2090 | 0,649436057 | 7,83760621 | 13,39623326 | 0,001233232 | 0,154865524 | EF2090 |
| EF2547 | 0,679862941 | 6,785911295 | 11,79881308 | 0,002741071 | 0,312608077 | EF2547 |
| EF2127 | 0,695658642 | 6,488922426 | 11,77304847 | 0,002776611 | 0,312608077 | EF2127 |
| EF2131 | 0,669370655 | 7,972770014 | 11,85345839 | 0,002667192 | 0,312608077 | EF2131 |
| EF2088 | 0,614282731 | 6,23226417 | 11,415698 | 0,003319806 | 0,361305528 | EF2088 |
| EF2087 | 0,606638731 | 6,716825496 | 10,6564003 | 0,004852796 | 0,511109048 | EF2087 |
| EF2138 | 0,603522163 | 7,436286948 | 10,07976814 | 0,006474499 | 0,660601213 | EF2138 |
| EF2129 | 0,598354065 | 8,545968075 | 9,935753497 | 0,006957906 | 0,688410977 | EF2129 |
| EF2139 | 0,597253932 | 8,596362884 | 9,551484377 | 0,008431824 | 0,791973807 | EF2139 |
| EF3087 | 0,484242779 | 5,674013726 | 9,53778788 | 0,008489765 | 0,791973807 | EF3087 |
| EF2135 | 0,593647869 | 7,21386294 | 9,143555797 | 0,010339561 | 0,937740711 | EF2135 |
| EF0081 | -0,187456786 | 9,238266884 | 8,977067091 | 0,01123711 | 0,945711336 | EF0081 |
| EF2137 | 0,605836033 | 6,367697197 | 8,915906971 | 0,01158605 | 0,945711336 | EF2137 |
| EF2113 | 0,577750922 | 6,8084834 | 8,941010143 | 0,011441536 | 0,945711336 | EF2113 |
| EF0228 | 0,49195471 | 9,283925189 | 9,000829259 | 0,011104391 | 0,945711336 | adk |
| EF2576 | 0,222105531 | 1,730785446 | 3,435500905 | 0,179469419 | 0,999742102 | EF2576 |
| EF2756 | 0,075959064 | 9,347407073 | 5,41733563 | 0,066625505 | 0,999742102 | dinP |
| EF2314 | 0,02431865 | 5,644803252 | 5,531969108 | 0,062914126 | 0,999742102 | EF2314 |
| EF2313 | -0,028266273 | 3,737176537 | 5,017293518 | 0,081378289 | 0,999742102 | EF2313 |
| EF1968 | 0,299719721 | 7,445817085 | 3,93369016 | 0,139897526 | 0,999742102 | EF1968 |
| EF1967 | 0,323836526 | 8,341091542 | 4,039434565 | 0,132692974 | 0,999742102 | EF1967 |
| EF1356 | 0,483931422 | 11,74024665 | 5,598106191 | 0,060867671 | 0,999742102 | lpdA |
| EF2038 | 0,077456481 | 3,597019743 | 2,886229531 | 0,236190932 | 0,999742102 | EF2038 |
| EF0202 | 0,199730083 | 8,125135377 | 3,197874062 | 0,202111242 | 0,999742102 | EF0202 |
| EF2318 | -0,00544583 | 6,950697488 | 4,271106364 | 0,118179198 | 0,999742102 | EF2318 |
| EF1355 | 0,515301485 | 11,68942773 | 5,982697387 | 0,05021966 | 0,999742102 | aceF |
| EF1308 | 0,175599918 | 12,61336391 | 2,934551891 | 0,230552669 | 0,999742102 | dnaK |
| EF1310 | 0,081665905 | 10,42160976 | 2,861562765 | 0,239122003 | 0,999742102 | dnaJ |
| EF0077 | -0,026399858 | 10,54895834 | 3,762458071 | 0,152402682 | 0,999742102 | EF0077 |
| EF0079 | -0,102113392 | 10,28043665 | 4,799608931 | 0,090735694 | 0,999742102 | EF0079 |
| EF3174 | -0,078282993 | 6,710887066 | 4,231628088 | 0,120535129 | 0,999742102 | EF3174 |
| EF1594 | 0,356116246 | 1,605264468 | 1,454631351 | 0,483204328 | 0,999742102 | EF1594 |
| EF0078 | -0,102509488 | 9,229367371 | 4,681126326 | 0,096273405 | 0,999742102 | EF0078 |
| EF1309 | -0,005982176 | 7,357077039 | 3,252582217 | 0,196657605 | 0,999742102 | EF1309 |
| EF1307 | 0,26520751 | 10,18714679 | 2,616830341 | 0,270248014 | 0,999742102 | grpE |
| EF2211 | 0,121157073 | 6,503347688 | 2,27221675 | 0,321066062 | 0,999742102 | EF2211 |
| EF0076 | 0,074789957 | 10,63610459 | 2,531344154 | 0,282049673 | 0,999742102 | EF0076 |
| EF2322 | 0,169837762 | 6,360905265 | 2,169124969 | 0,338049655 | 0,999742102 | EF2322 |
| EF0767 | 0,117835837 | 8,836877701 | 2,232720832 | 0,327469481 | 0,999742102 | EF0767 |
| EF1354 | 0,441721505 | 11,38857893 | 4,407286302 | 0,11040022 | 0,999742102 | pdhB |
| EF2337 | 0,079954762 | 3,186644488 | 1,639305203 | 0,440584686 | 0,999742102 | EF2337 |
| EF2360 | 0,310687168 | 1,965870429 | 1,359853211 | 0,506654177 | 0,999742102 | EF2360 |
| EF2070 | 0,222586334 | 9,187039326 | 2,197647932 | 0,333262782 | 0,999742102 | mnmA |
| EF2633 | 0,073350338 | 12,05174617 | 2,298249626 | 0,316914007 | 0,999742102 | groEL |
| EF1082 | 0,11204285 | 6,539451201 | 2,056667685 | 0,357602286 | 0,999742102 | EF1082 |
| EF1830 | 0,051143445 | 5,268253353 | 2,208837502 | 0,331403454 | 0,999742102 | EF1830 |
| EF1522 | 0,177053919 | 9,330608284 | 2,046898241 | 0,359353347 | 0,999742102 | sigA |
| EF1085 | 0,190270431 | 7,844785999 | 1,966113193 | 0,374165676 | 0,999742102 | EF1085 |
| EF1368 | -0,088144079 | 10,38112365 | 3,583288086 | 0,166685905 | 0,999742102 | EF1368 |
| EF2332 | 0,196766319 | 5,505869305 | 1,78101814 | 0,410446753 | 0,999742102 | EF2332 |
| EF1521 | 0,182980242 | 9,635673031 | 1,877749159 | 0,391067703 | 0,999742102 | dnaG |
| EF1591 | 0,032659804 | 4,609965035 | 1,940139814 | 0,379056538 | 0,999742102 | EF1591 |
| EF3239 | 0,260190936 | 5,772938546 | 2,023078149 | 0,363658851 | 0,999742102 | EF3239 |
| EF2355 | 0,084966986 | 11,1260528 | 1,947475588 | 0,377668749 | 0,999742102 | clpB |
| EF1084 | 0,123938543 | 8,585783711 | 1,760575561 | 0,414663562 | 0,999742102 | EF1084 |
| EF2384 | 0,098218547 | 2,559148743 | 1,059108544 | 0,588867386 | 0,999742102 | EF2384 |
