# Supplementary material for: Phage-mediated Dispersal of Biofilm and Distribution of Bacterial Virulence Genes Is Induced by Quorum Sensing
Source: PLoS Pathog. 2015 Feb 23;11(2):e1004653. doi: 10.1371/journal.ppat.1004653 (PMC4338201; doi:10.1371/journal.ppat.1004653)
Supplement: S1 Fig — (DOCX) [file ppat.1004653.s004.docx]

**Figure S1: Determination of the AI-2 concentration produced by *E. faecalis* V583ΔABC*pp5-*:** The concentration of AI-2 in filter-sterilized supernatants of *E. faecalis*ΔABC*pp5-* was determined by a FRET-based AI-2 assay [27,28]. Briefly, the reporter protein consisting of CFP, LuxP, and YFP (CLPY) was produced in an AI-2 *E. coli* strain, purified by Ni-NTA affinity chromatography and used for the assay in a final concentration of 0.025 mg/ml in reaction buffer (25 mM sodium phosphate buffer pH 8.0, 35 mM sodium chloride and 1 mM boric acid). The fluorescence emission measured at 540 nm and 485 nm upon excitation at 430 nm gives the FRET ratio (YFP/CFP). A calibration curve for AI-2 (1-60 µM) was used to determine the concentration of AI-2 in the supernatants [29,30]. All experiments were done in triplicate at different time points. As seen for *E. faecalis* V583ΔABC, at 5 to 6 hours the AI-2 concentration secreted by the *p5-* mutant peaked at a maximum of about 80 µM and then slowly decreased. The error bars represent standard error of the mean.
