# Supplementary material for: Phage-mediated Dispersal of Biofilm and Distribution of Bacterial Virulence Genes Is Induced by Quorum Sensing
Source: PLoS Pathog. 2015 Feb 23;11(2):e1004653. doi: 10.1371/journal.ppat.1004653 (PMC4338201; doi:10.1371/journal.ppat.1004653)
Supplement: S2 Fig — (DOCX) [file ppat.1004653.s005.docx]

 **Fig. S2: Determination of the AI-2 concentration in the supernatants of *E. faecalis* V583ΔABC after supplementation with 100 µM AI-2:** To address the question whether *E. faecalis* produces AI-2 also when exogenous AI-2 is added, we measured the AI-2 concentration in absence or presence of the addition of 100 µM AI-2 over an eight hour time periode. The same AI-2 (purchased from OMM scientific) used for the calibration curve was used for supplementation. This calibration curve was also used to measure the concentration of AI-2 in supernatants of *V. harveyi*. When exogenous AI-2 at a concentration of 100 µM was added to a growing culture, initial values reached 100 µM and increased up to 130 µM within the first hour. Afterwards the AI-2 concentration drops over time, indicating that bacteria sense the AI-2 in the culture environment and stop endogenous production accordingly. When no exogenous AI-2 is added, the amount produced in the culture reaches about 100 µM after 6 hours Error bars represent standard error of means.
