# Supplementary material for: Phage-mediated Dispersal of Biofilm and Distribution of Bacterial Virulence Genes Is Induced by Quorum Sensing
Source: PLoS Pathog. 2015 Feb 23;11(2):e1004653. doi: 10.1371/journal.ppat.1004653 (PMC4338201; doi:10.1371/journal.ppat.1004653)
Supplement: S3 Fig — Panel A shows biofilm formation without supplementation of AI-2. Panel B shows biofilm formation after supplementation with AI-2 of E. faecalis V583 ΔABC. (DOCX) [file ppat.1004653.s006.docx]

**
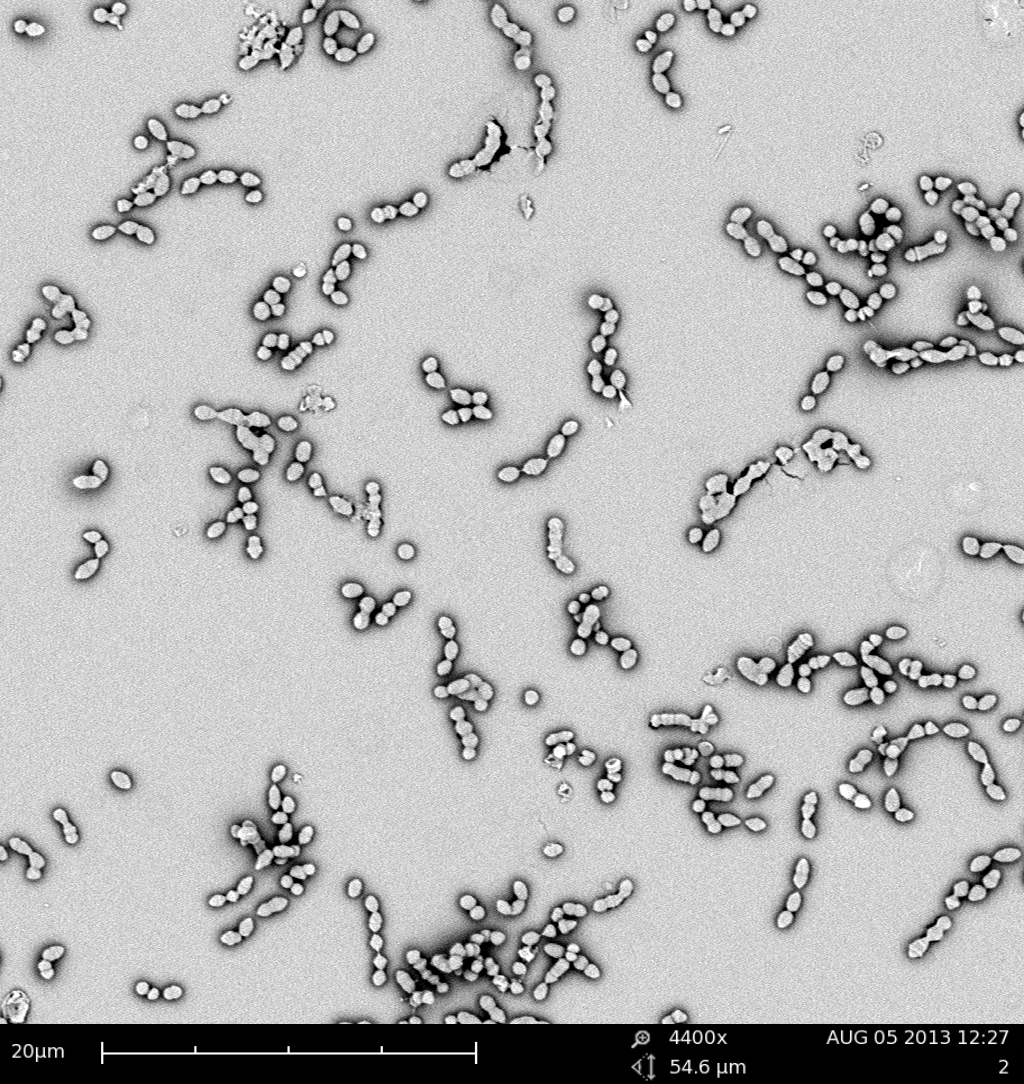

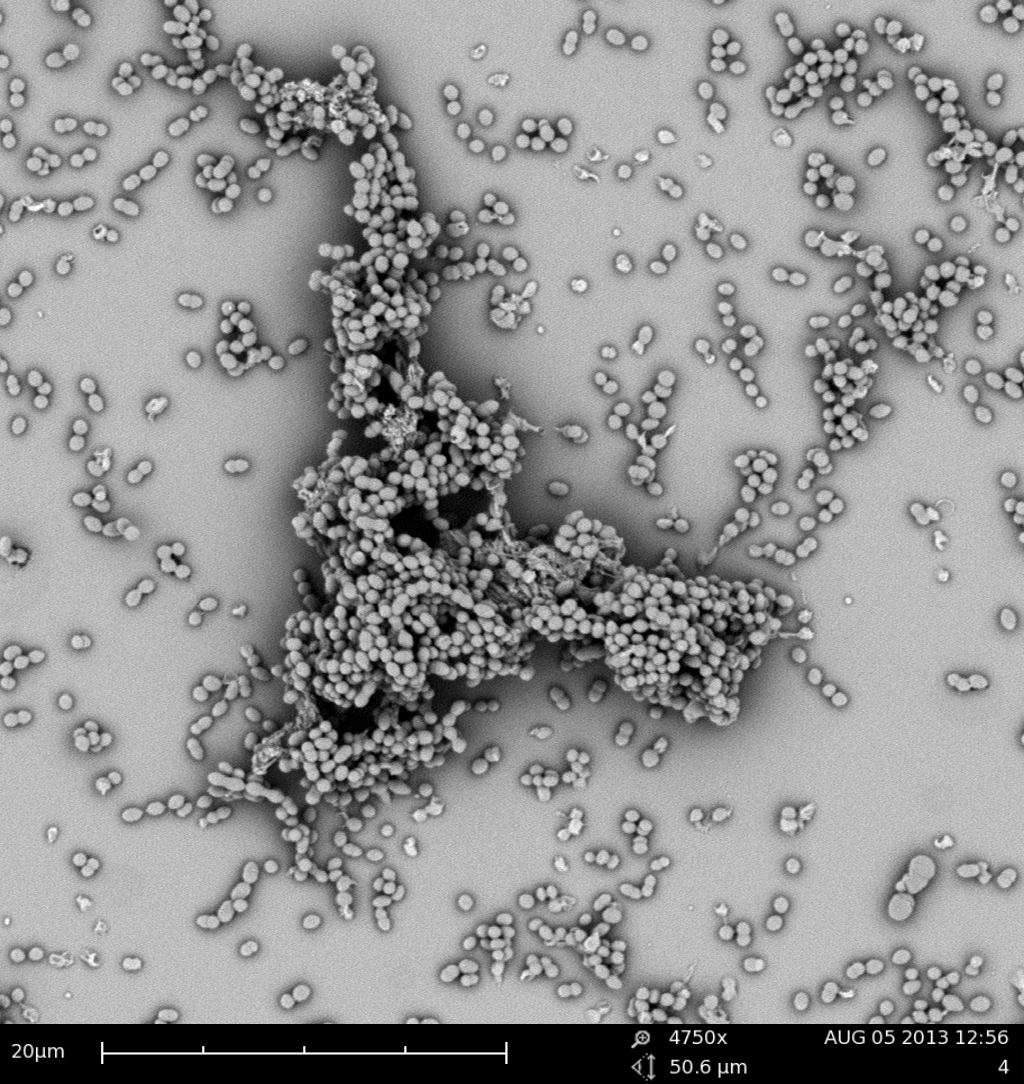
A B**

**+ 100 µM AI-2**

**Fig. S3**: **Scanning microscopy slides showing *E. faecalis* V583ΔABC grown without AI-2 (A) and with 100 μM AI-2 (B).** Biofilms grown on poly-L-lysine-coated glass slides were treated with no (A) or 100 µM AI-2 (B) in growth medium in 12-well-plates and carefully washed to remove medium. Samples were fixed with glutaraldehyde and serially dehydrated. The glass slides were air-dried overnight at room temperature and placed on specimen mounts. Slides were coated with 80% Pt-20% Pd to 4 nm using a Cressington 208HR sputter coater at 40 mA prior to examination with a Phenom Table-top scanning electron microscope.

**Panel A:** *E. faecalis* V583ΔABC grown without AI-2 shows aggregation and clustering of cells.

**Panel B:** Compared to *E. faecalis* V583ΔABC grown without AI-2, a significant decrease in biofilm formation was observed when 100 µM AI-2 was added.
