# Supplementary material for: Phage-mediated Dispersal of Biofilm and Distribution of Bacterial Virulence Genes Is Induced by Quorum Sensing
Source: PLoS Pathog. 2015 Feb 23;11(2):e1004653. doi: 10.1371/journal.ppat.1004653 (PMC4338201; doi:10.1371/journal.ppat.1004653)
Supplement: S4 Fig — (DOCX) [file ppat.1004653.s007.docx]

**Fig. S4: Growth curve of *E. faecalis* V583** ΔABC **in absence or presence of 100 µM AI-2 was measured at an optical density of 630nm.** Both curves show equally growth independent if AI-2 is added or not.
