# Supplementary material for: Phage-mediated Dispersal of Biofilm and Distribution of Bacterial Virulence Genes Is Induced by Quorum Sensing
Source: PLoS Pathog. 2015 Feb 23;11(2):e1004653. doi: 10.1371/journal.ppat.1004653 (PMC4338201; doi:10.1371/journal.ppat.1004653)
Supplement: S5 Fig — Panel A shows viable cell numbers during biofilm formation of E. faecalis V583ΔABC. Panel B demonstrates viable number of Symbioflor 1 and transduced Symbioflor 1 during biofilm formation. (DOCX) [file ppat.1004653.s008.docx]

**A B**

**Fig. S5: Bacterial survival of *E. faecalis* V583ΔABC (panel A), Symbioflor 1, Symbioflor transduced with prophage5 and polylysogenic Symbioflor (prophage 1, 2, 5 and 7) (panel B).** To study the relative living cell number Symbioflor strains were grown in Petri dishes and collected after 1, 6 and 24 hours by scraping attached bacteria. The total number of bacteria was determined using a Thoma counting chamber and in parallel, bacteria were plated on TSA plates to quantify the number of colony forming units. Finally, bacterial cell survival was calculated as follows: cfu / total cell number * 100, to obtain results in percentage.

**Panel A:** After 18h of incubation, no significant differences were seen between viable cell numbers between cultures grown with or without AI-2. At later time points (e.g. 24h and 30h) higher cell counts were observed in cultures without AI-2, indicating that lysis of bacteria by phages may be responsible for this effect.

**Panel B:** Bacterial survival of Symbioflor 1 was compared to polylysogenic strains transduced with either pp5 or pp1, pp5 and pp7. Again, at later timepoints (e.g. 6h and 24h) the transduced strains showed a significant decrease in viable counts when 100 µM AI-2 was added.
