# Supplementary material for: Phage-mediated Dispersal of Biofilm and Distribution of Bacterial Virulence Genes Is Induced by Quorum Sensing
Source: PLoS Pathog. 2015 Feb 23;11(2):e1004653. doi: 10.1371/journal.ppat.1004653 (PMC4338201; doi:10.1371/journal.ppat.1004653)
Supplement: S6 Fig — (DOCX) [file ppat.1004653.s009.docx]

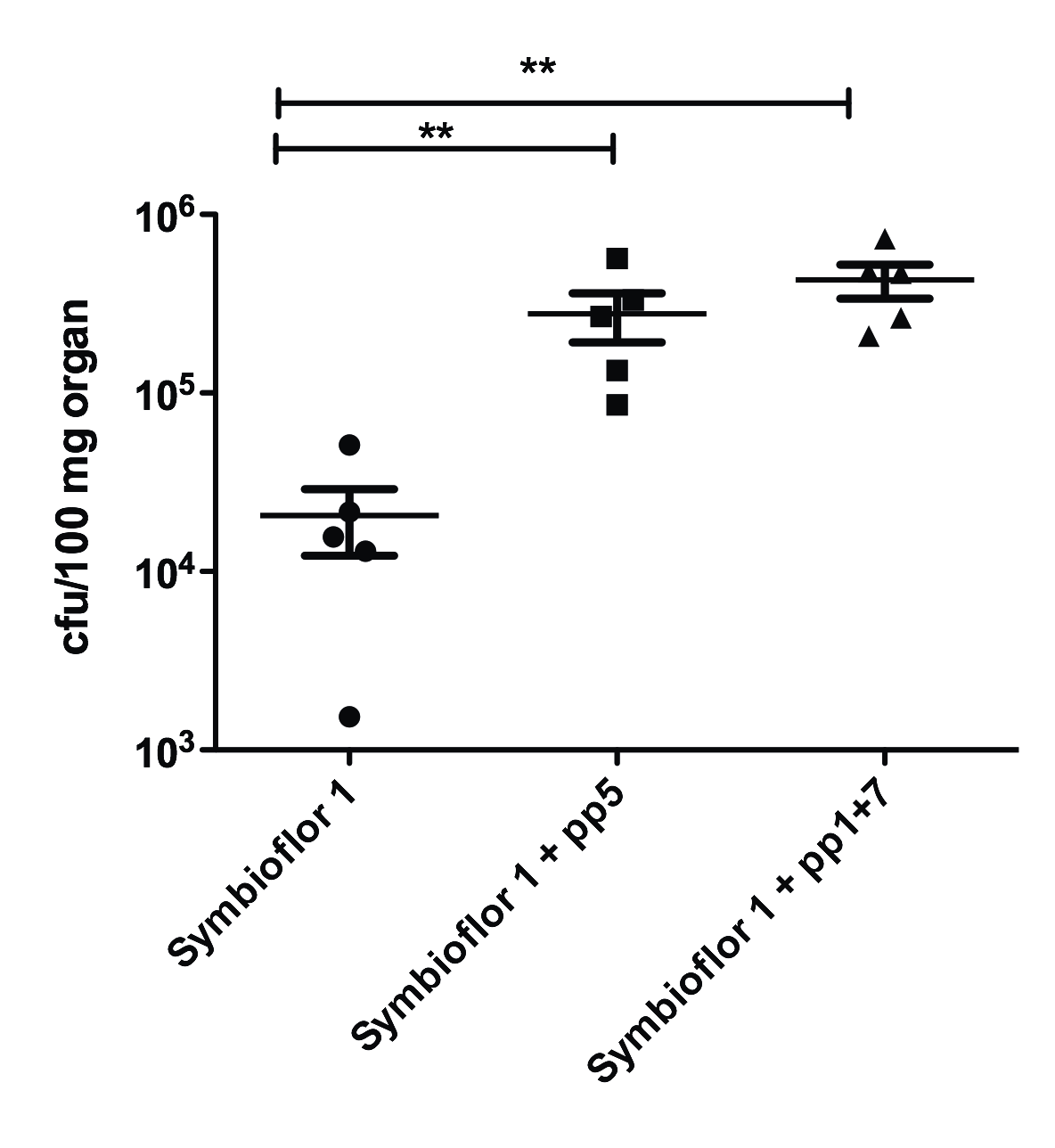


**Fig. S6: Mouse bacteremia model comparing Symbioflor wild-type and transduced Symbioflor strains:** The virulence of probiotic *E. faecalis* Symbioflor 1, *E. faecalis* Symbioflor 1 + pp5 and *E. faecalis* Symbioflor 1 pp1 + pp7 was compared in a mouse bacteremia model. Five female BALB/c mice 6-8 weeks old were infected by i.v. injection of *E. faecalis* Symbioflor 1 (9.9 x 10^7^ cfu) or transduced *E. faecalis* Symbioflor 1 (8.0 x 10^7^ cfu) via the tail vein. Twenty-four hours after infection, mice were sacrificed and bacterial counts were enumerated by serial dilutions on TSA plates. Statistical significance was assessed by Mann-Whitney test. In comparison to probiotic *E. faecalis* Symbioflor 1, transduced *E. faecalis* Symbioflor 1 strains show significantly higher colony forming units and in particular *E. faecalis* Symbioflor 1 transduced by prophage 5. Presence of prophage 5 in the genome was comfirmed by PCR after isolating the bacteria from the mice organs. As prophage 7 requires prophage 1 as a helper phage for encapsidation, we tested prophage 1 and 7 together in one animal set up. Statistical analysis was determined by Mann Whitney and ** indicates p<0.01.
